# The case of a 61‐year‐old man with unusual headaches

**DOI:** 10.1002/acn3.51179

**Published:** 2020-12-18

**Authors:** Margaret Yu, Sarah Brooker, Shubadra Priyadarshini

**Affiliations:** ^1^ Northwestern University Chicago Illinois USA

## Abstract

A 61‐year‐old man with past medical history significant for prediabetes, hyperlipidemia and high‐grade prostate intraepithelial neoplasia presents with headaches for one month. Imaging of his brain reveals hydrocephalus and spine imaging reveals a cord lesion. These findings are discussed further in the case.

## Diagnosis

Intramedullary spinal cord lesion complicated by subarachnoid hemorrhage with communicating hydrocephalus

## Take‐Home Points:

**Figure 1:**
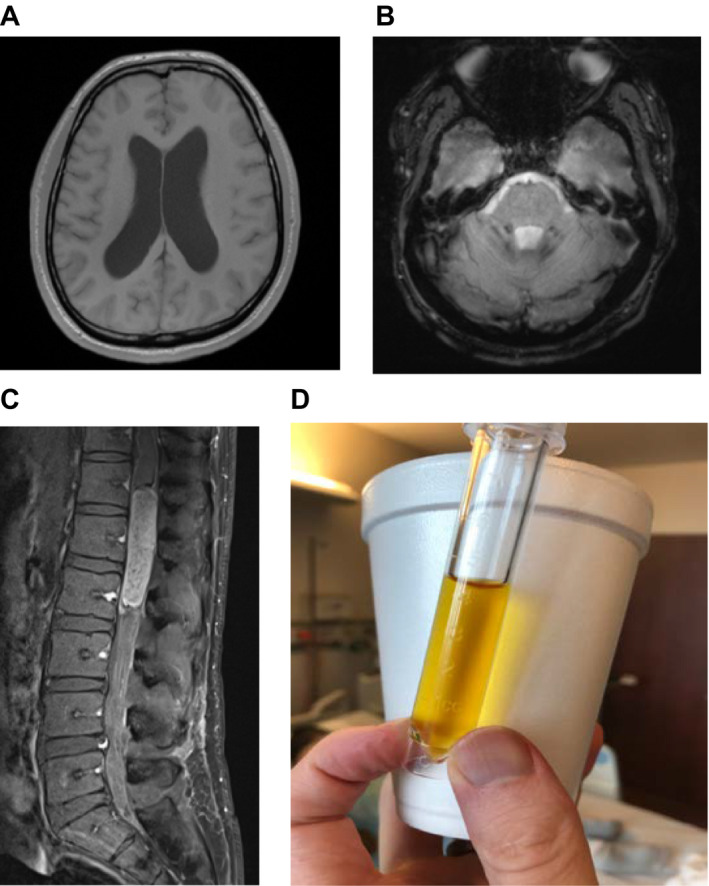
A. T1 MRI Axial of brain demonstrating hydrocephalus B. GRE MRI Axial of brain demonstrating hemosiderin deposits in the bilateral cerebellar hemispheres concerning subarachnoid hemorrhage C. Intramedullary mass demonstrated on sagittal MRI lumbar spine D. Cerebrospinal fluid from lumbar puncture demonstrating xanthrochromia.

- Hydrocephalus has a wide array of potential etiologies. The most important thing is to first determine if the hydrocephalus is communicating or noncommunicating. Hydrocephalus can be congenital or acquired. Common acquired causes of hydrocephalus include infectious, post‐hemorrhagic, and secondary to mass effect.
- Spinal cord lesions can be intramedullary or extramedullary as well as intradural or extradural. The differential for each of those is different. The most common intramedullary lesion in the adult is an ependymoma, especially myxopapillary ependymoma in the conus/filum terminale.
- The Nonne‐Froin sign is a manifestation of spinal tumors causing partial or complete spinal canal blockage. It manifests as xanthrochromia, high protein content, and coagulation of the CSF (Figure 1).

